# Targeted-Deletion of a Tiny Sequence via Prime Editing to Restore SMN Expression

**DOI:** 10.3390/ijms23147941

**Published:** 2022-07-19

**Authors:** Miaojin Zhou, Shuqing Tang, Nannan Duan, Mi Xie, Zhuo Li, Mai Feng, Lingqian Wu, Zhiqing Hu, Desheng Liang

**Affiliations:** Center for Medical Genetics, School of Life Sciences, Central South University, Changsha 410078, China; zhoumiaojin@sklmg.edu.cn (M.Z.); tangshuqing@sklmg.edu.cn (S.T.); duannnjj@163.com (N.D.); xiemi@sklmg.edu.cn (M.X.); lizhuo@sklmg.edu.cn (Z.L.); fengmai@sklmg.edu.cn (M.F.); wulingqian@sklmg.edu.cn (L.W.)

**Keywords:** spinal muscular atrophy, survival motor neuron gene 2, intronic splicing silencer-N1, prime editing, targeted-deletion, induced pluripotent stem cells, motor neurons, endoplasmic reticulum stress

## Abstract

Spinal muscular atrophy (SMA) is a devastating autosomal recessive motor neuron disease associated with mutations in the survival motor neuron 1 (*SMN1*) gene, the leading genetic cause of infant mortality. A nearly identical copy gene (*SMN2*) is retained in almost all patients with SMA. However, *SMN2* fails to prevent disease development because of its alternative splicing, leading to a lack of exon 7 in the majority of *SMN2* transcripts and yielding an unstable truncated protein. Several splicing regulatory elements, including intronic splicing silencer-N1 (ISS-N1) of *SMN2* have been described. In this study, targeted-deletion of ISS-N1 was achieved using prime editing (PE) in SMA patient-specific induced pluripotent stem cells (SMA-iPSCs) with a high efficiency of 7/24. FL-SMN expression was restored in the targeted-deletion iPS clones and their derived motor neurons (iMNs). Notably, the apoptosis of the iMNs, caused by the loss of SMN protein that leads to the hyperactivity of endoplasmic reticulum (ER) stress, was alleviated in targeted-deletion iPSCs derived-iMNs. Thus, this is the first study to demonstrate that the targeted-deletion of ISS-N1 via PE for restoring FL-SMN expression holds therapeutic promise for SMA.

## 1. Introduction

Spinal muscular atrophy (SMA) is one of the most common inherited causes of newborn mortality, with an occurrence of 1–2/10,000 and a carrier frequency of 1/40–1/60 [1,2,3]. Infants with the most severe forms of type I SMA and disease phenotype appearing in the early postnatal period usually die before 2 years of age because of respiratory failure if no intervention is provided [4,5]. SMA is caused by homozygous deletions in the survival motor neuron gene 1 (*SMN1*), which significantly reduces SMN protein levels and leads to SMA-specific changes in motor neurons, including hyper-activation of the endoplasmic reticulum (ER) stress pathway [6], resulting in the loss of lower α-motor neurons in the spinal cord [7,8].

*SMN2*, an almost identical copy of *SMN1*, is present in the same region of chromosome 5q in the human genome. No less than one copy of *SMN2* is present in almost all patients with SMA, and mutations in *SMN2* have not been found to have clinical consequences if *SMN1* is retained [9,10]. However, *SMN2* fails to compensate for the loss of *SMN1* because of a translationally silent C-to-T transition at the sixth position of exon 7 (c.840 C to T) via alternative splicing; consequently, ~90% of *SMN2*-derived transcripts lack exon 7 (Δ7-SMN), and a truncated and unstable protein forms. Only ~10% of full-length SMN mRNA (FL-SMN) is produced from *SMN2* and can be translated into a fully functional SMN protein [11]. Therefore, strategies that increase *SMN2*-derived FL-SMN expression have been investigated and approved for clinical applications.

The pre-mRNA of *SMN* exon 7 is accurately spliced through a complex interaction between positive and negative regulatory factors. Several important splicing regulators, including the splicing activator SF2/ASF (whose binding site is within exon 7) [12], the splicing repressor hnRNPA1 [13], and serine/arginine (SR)-like protein Tra2β1 [14], have been identified. The substitution of c.840 C to T destroys the binding site of the splicing activator and creates an hnRNPA1 binding site. hnRNPA1 binding to intronic splicing silencer-N1 (ISS-N1) can repress *SMN2* exon 7 splicing [9,12]. Several therapeutic approaches have been used to improve SMN protein levels by blocking ISS-N1, including antisense oligonucleotides (ASOs) [15] and disruption with CRISPR/Cas9 [16]. However, ASOs require repeated intrathecal injections, and CRISPR/Cas9 editing through error-prone repair leads to byproducts production [16].

Prime editing (PE), a new “search-and-replace” genome editing system, combines a Cas9 nickase (H480A) with an engineered reverse transcriptase, which enables efficient base substitution, insertion, and deletion without requiring double-strand breaks or donor DNA templates [17].

In this study, a genome editing strategy was developed through which the PE targeted-deletion of a short ISS-N1 sequence disrupted splicing regulatory elements. Thus, the inclusion rates of *SMN2* exon 7 increased, and FL-SMN expression was restored in targeted-deletion induced pluripotent stem cells (iPSCs) and their derived motor neurons (iMNs). Moreover, the degeneration of these iMNs was ameliorated when treated with ER stress.

## 2. Results

### 2.1. Designing and Screening of an Efficient ISS-N1-Targeted PE System

**Figure 1:**
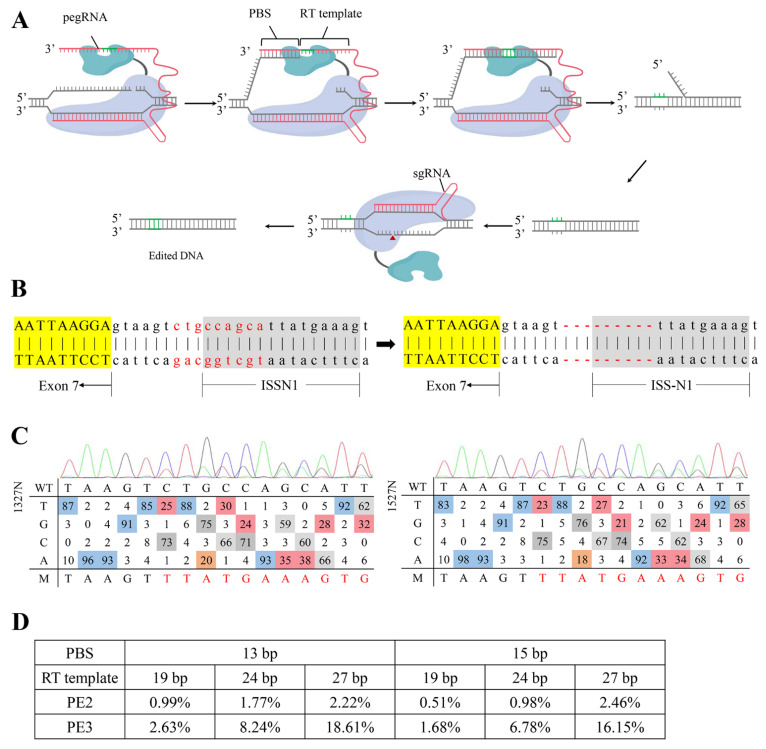
Site-specific Prime editing system design and activity assay. (**A**) The schematic diagram of the Prime editing system. The PE2 system is mediated by SpCas9 (H840A) nickase fused to an engineered reverse transcriptase and a prime editing guide RNA (pegRNA). And the PE3 system uses an additional nicking single guide RNA (sgRNA) to nick the opposite strand of pegRNA. PBS, primer binding site; RT template, reverse transcription template. (**B**) Schematic representation of the in situ targeted-deletion. The deleted bases are typed in red. The deletions are indicated by a red dashed line. Yellow-shaded bases are sequences of *SMN2* exon 7, and gray-shaded bases are ISS-N1 sequences. (**C**) Estimated editing frequency using EditR after Sanger sequencing. The top curve is the chromatogram of the protospacer. A, green. T, red. C. blue. G, black. The bottom panel shows the percentage of signal area for the corresponding base (ACGT) at each position. Bases of non-noise signal are colored in squares. From red to blue, the corresponding base signal percentage gradually increases. Positions have one square colored in indicate that only one peak being present at that position. WT, wild type. M, mutation. (**D**) Targeted deep sequencing analysis of the editing efficiency of PE2 or PE3 at the ISS-N1 using pegRNAs containing 13 or 15 nt PBS and RT template (19 nt, 24 nt, and 27 nt) in HEK-293T.

The scheme of the PE system is illustrated in Figure 1A. The system is composed of a nick spCas9 (H840A), a reverse transcriptase, an engineered PE guide RNA (pegRNA) containing an sgRNA scaffold, a reverse transcription (RT) template, and a primer-binding sequence (PBS). Under the guidance of the pegRNA, the target DNA was searched and nicked via PE, and the genetic information encoded by the pegRNA was extended into the genome via RT. A nicking single guide RNA (sgRNA) targeted the opposite strand of pegRNA to improve the integration of edits into the genome, which was referred to as PE3 [17].

The direct blocking of the core of the antisense target (cugccagc) at positions 7–14 of intron 7 promoted the inclusion of exon 7 in *SMN2* [18]. The designation of pegRNA was restricted by the protospacer adjacent motif (PAM) sequence, and the downstream of the nicked site was modified via PE. Therefore, a 9 nt sequence containing the core of the antisense target of ISS-N1 was deleted (Figure 1B). One nicking sgRNA and six pegRNAs with different lengths of PBS (13 and 15 nt) and RT template (19 nt, 24 nt, and 27 nt) were designed and constructed. Each pegRNA was co-transfected with or without nicking sgRNA into HEK-293T cells to evaluate the targeted-deletion efficiency. The genomic region encompassing the target site was then amplified and sequenced. The targeted-deletion efficiency was analyzed via EditR (https://moriaritylab.shinyapps.io/editr_v10/, accessed on 4 May 2020) (Figure 1C) [19] and targeted deep sequencing. The results showed that the targeted-deletion efficiency increased with the extension of the RT template and was higher than that of PE2. Furthermore, 1327N (PBS 13 nt, RT template 27 nt, and co-transfected with nicking sgRNA) and 1527N (PBS with 15 nt, RT template 27 nt, and co-transfected with nicking sgRNA) had high targeted-deletion efficiencies of 18.61% and 16.15%, respectively (Figure 1D).

### 2.2. Targeted-Deletion ISS-N1 of SMN2 in SMA-iPSCs by PE

**Figure 2:**
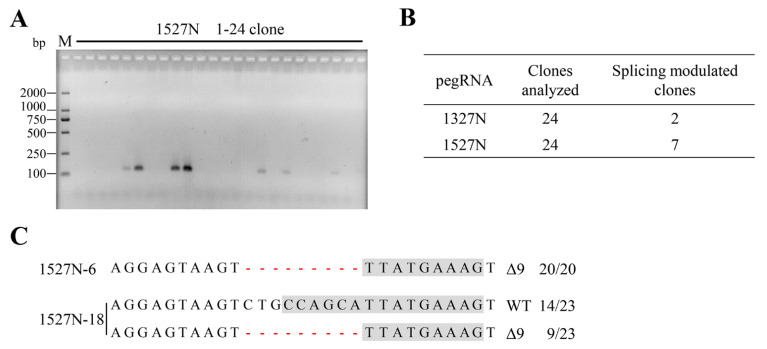
Targeted-deletion and screening. (**A**) Twenty-four candidate mono-clones of 1527N were picked, followed by genomic DNA extraction and PCR analysis using a specific primer. The targeted-deletion obtained a 121 bp band. M, DL2000 DNA ladder. (**B**) Splicing-modulation efficiencies by targeting ISS-N1. (**C**) T-A cloning and sequencing of the positive clones. The deletions are indicated by a red dashed line. The column on the right indicates the percent of the relevant genotype in total sequencing reads. For 1527N-6, all the 20 reads were 9-nt deleted, which indicated 1527N-6 has three targeted-deletion copies. 1527N-18 has 14 reads with WT genotype and 9 reads with deleted 9-nt, indicating one targeted-deletion copy with two unedited copies. WT, wild-type. Δ, deletion.

We genotyped and identified an SMA patient with three copies of *SMN2* and homozygous deletion of exons 7 and 8 of *SMN1*. An iPSC line (SMA-iPSCs) derived from urine cells of the patient with SMA was established [20]. SMA-iPSCs harbored the deletion of exons 7 and 8 of *SMN1* and maintained a normal karyotype. The targeted-deletion of SMA-iPSCs was performed via nucleofection using 1327N or 1527N. For each transfection, 24 clones were picked up, expanded, and screened via PCR with the specific primer F1/R1. Of the 24 clones, 2 (8.33%) for 1327N and 7 (29.17%) for 1527N were positive for the specific band of 121 bp (Figure 2A,B and Figure S1A). Mono-clone genomic DNA was isolated, and the encompassing region was PCR-amplified, cloned, and sequenced to analyze whether these mono-clones had indels in the targeted-deletion copy of *SMN2*. No less than 20 reads were analyzed. All mono-clones contained at least one targeted-deletion copy (Figure 2C and Figure S1B). Two stably targeted-deletion clones were selected for further research: 1527N-6, which had three edited ISS-N1 copies, and 1527N-18, which had one targeted-deletion copy. These results revealed that targeted-deletion was efficiently achieved. Moreover, 1527N-6 and 1527N-18 expressed pluripotency markers and maintained a normal karyotype (Figure S2), indicating that targeted-deletion of ISS-N1 of *SMN2* did not affect pluripotency.

Specific primers (Table S1) were selected and designed to test the specificity of pegRNA and nicking sgRNA used in the experiment and amplify 19 putative off-target sites, 9 sites for pegRNA (Table S2), and 10 sites for nicking sgRNA (Table S3), which were predicted by the CCTop-CRISPR/Cas9 target online predictor (https://cctop.cos.uni-heidelberg.de:8043/, accessed on 20 July 2020) [21]. After genomic DNA was isolated from SMA-iPSCs and targeted-deletion iPSCs (1527N-6 and 1527N-18), the potential off-target sites were amplified and sequenced (Table S4). No indels were observed at any of the examined loci (Figure S3).

### 2.3. SMN Expression in Targeted-Deletion iPSCs Clones

**Figure 3:**
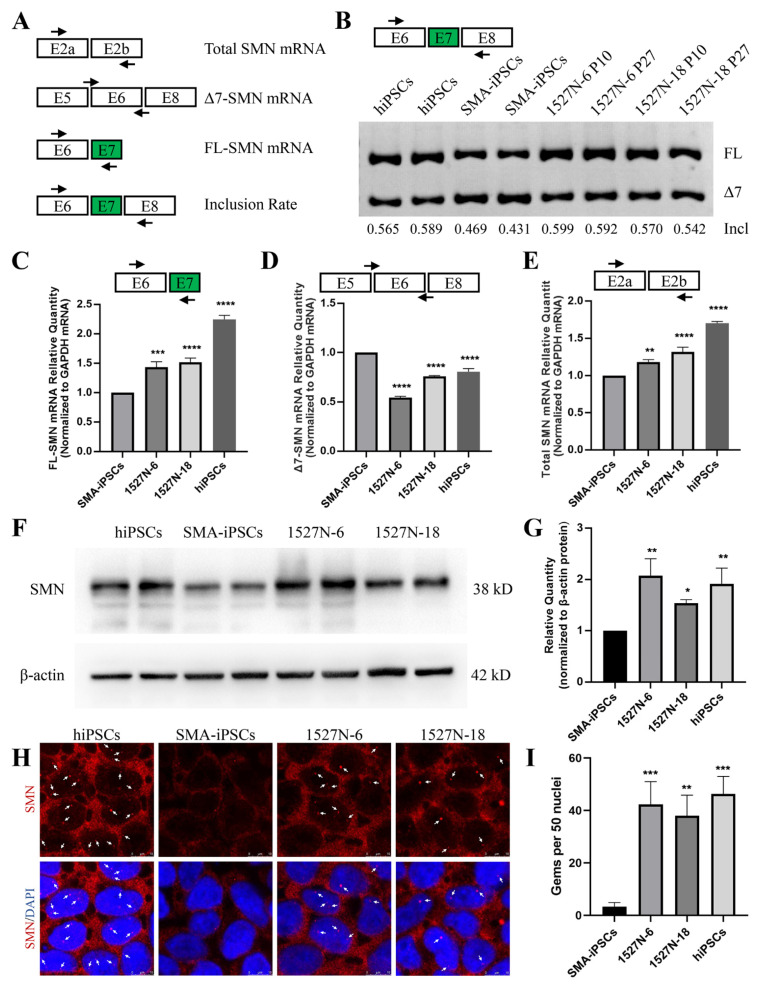
Restored SMN expression in iPSCs. (**A**) Schematic representation of the locus of the four primer pairs. E, exon. (**B**) RT-PCR analysis of *SMN2* mRNA in hiPSCs, SMA-iPSCs, and targeted-deletion iPSCs. The targeted-deletion iPSCs were evaluated in passages (P) 10 and 27. Incl = FL/(FL + ∆7). (**C**–**E**) qRT-PCR analyzed the expression of FL-SMN, Δ7-SMN, and total SMN mRNA. *GAPDH* was used as the internal reference. Bars represent the Mean ± standard error of the mean (SEM); *n* = 3. ** *p* < 0.01. *** *p* < 0.001. **** *p* < 0.0001. (**F**) The SMN protein expression levels in hiPSCs, SMA-iPSCs, and targeted-deletion iPSCs (1527N-6 and 1527N-18). Β-actin was used as the internal reference. (**G**) Values for each sample were normalized to β-actin protein levels. Data shown indicate mean ± standard deviation (SD). * *p* < 0.05. ** *p* < 0.01. (**H**) Immunofluorescence of SMN in hiPSCs, SMA-iPSCs, and targeted-deleted iPS cell lines. The red fluorescence shows SMN protein, and the gems were pointed out with white arrows. DAPI was used to visualize the nucleus. (**I**) The number of gems was more in the hiPSCs and targeted-deletion iPSCs compared with SMA-iPSCs. Data shown indicate mean ± SD. ** *p* < 0.01. *** *p* < 0.001.

The SMN expression in targeted-deletion iPSCs was evaluated. Four primer pairs were used to detect SMN transcripts (Figure 3A). The exon 7 inclusion rate was detected via RT-PCR, which showed that the inclusion rate in targeted-deletion clones was higher than that in SMA-iPSCs and similar to that in human iPSCs (hiPSCs; Figure 3B), which was generated and genotyped in our previous study [20]. The transcripts of FL-SMN, total SMN, and Δ7-SMN were analyzed using quantitative RT-PCR (qRT-PCR), with glyceraldehyde-3-phosphate dehydrogenase (GAPDH) transcripts used as internal controls. The FL-SMN and total SMN transcription levels in targeted-deletion iPSCs were significantly higher than those in SMA-iPSCs (Figure 3C,D). The mRNA levels of Δ7-SMN were lower than those of SMA-iPSCs (Figure 3E). Cell lysates were collected to validate SMN protein levels, and western blotting was performed to detect SMN protein. Normal hiPSCs were used as positive controls. SMN expression in 1527N-6 and 1527N-18 was similar to that in hiPSCs and significantly higher than in SMA-iPSCs (Figure 3F,G and Figure S1C). As SMN sub-nuclear bodies (also called gems) are usually deficient in SMA patient cells, immunostaining was performed to test whether the increased SMN expression after targeted-deletion could induce nuclear accumulation of gems. The SMN protein was present in the nucleus and cytoplasm in all iPS cell lines. 1527N-6 and 1527N-18 had a profound increase in the number of gem structures, while few SMN gems were observed in SMA-iPSCs (Figure 3H,I). Therefore, only one edited ISS-N1 among the three *SMN2* loci was sufficient to correct splicing in SMA-iPSCs.

### 2.4. Restoration of SMN Expression in Targeted-Deletion iPSC-Derived MNs

**Figure 4:**
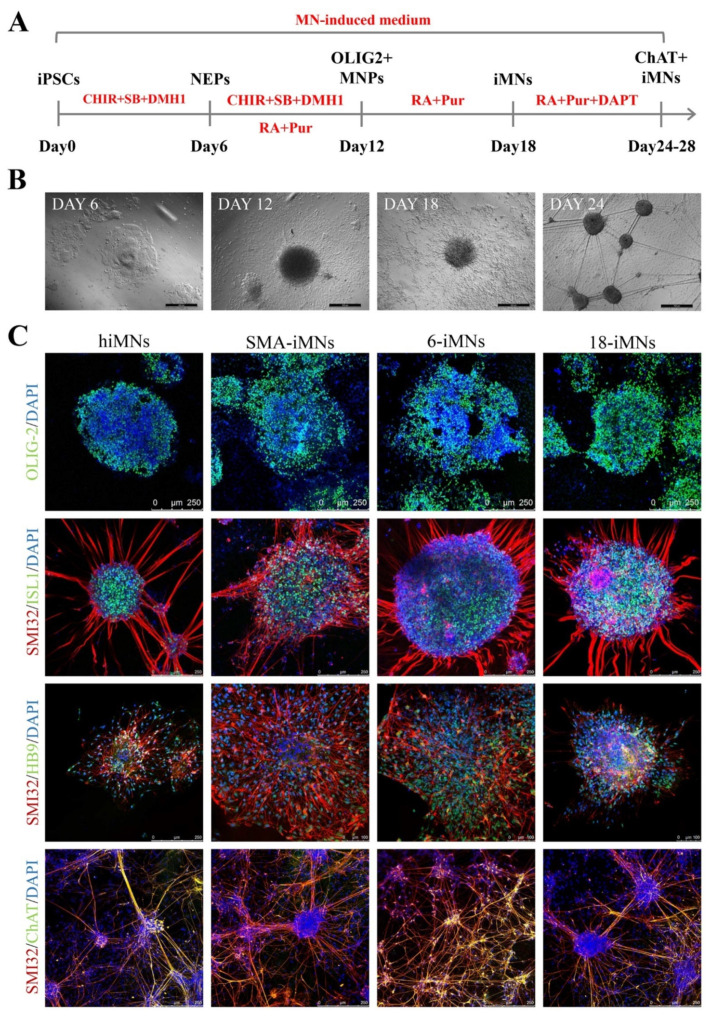
Differentiation of iPSCs into motor neurons. (**A**) Schematic protocol for motor neuron differentiation. NEPs, neuroepithelial progenitors; MNPs, motor neuron progenitors; iMNs, induced motor neurons; CHIR, CHIR99021; SB, SB431542; RA, retinal acid. (**B**) Dynamic changes in cellular morphology during differentiation of iPSC-derived MNs (iMNs). Scale bar: 500 μm. (**C**) Immunostaining of MNPs markers OLIG-2 at day 12 and motor neuron markers (ISL1, SMI32, HB9 at day 18, and ChAT, SMI32 at day 24). DAPI was used for nuclear staining. ISL1, ISLET1.

MNs are the most affected cell type of SMA. In this study, 1527N-6, 1527N-18, SMA-iPSCs, and hiPSCs were differentiated into MNs (6-iMNs, 18-iMNs, SMA-iMNs, and hiMNs) via a combination of small molecules regulating multiple signaling pathways to further validate whether the FL-SMN expression was restored in MN by using a previously described method (Figure 4A) [20]. Cell morphology changed dynamically during differentiation (Figure 4B). After 12 days of continuous differentiation, an abundant population of OLIG2-positive motor neuron progenitors (MNPs) was obtained. After six days of culture with a high retinoic acid (RA) concentration and a low sonic hedhehog (SHH) signaling agonist concentration, almost pure ISLET1+/SMI32+ cells were harvested, and all cell lines expressed the MN-specific marker HB9. Subsequently, a NOTCH signal inhibitor was added to the differentiation system. After 6–10 days, all iMNs cell lines were positive for choline acetyltransferase (ChAT) and SMI32 (Figure 4C). During MN maturation, it was found that the SMA-iMNs could hardly survive at low density, while there was no difference between 6-iMNs and 18-iMNs with hiMNs.

**Figure 5:**
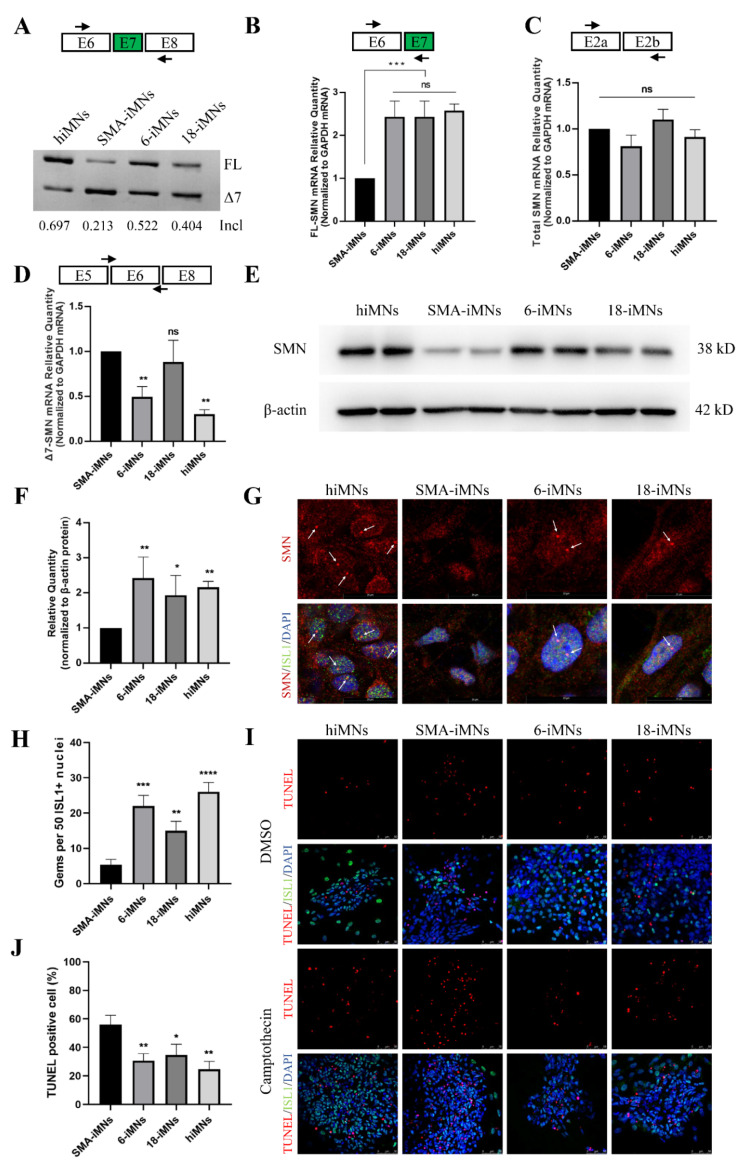
SMN expression in iMNs. (**A**) RT-PCR analysis of *SMN2* mRNA in iMNs at day 28. (**B**–**D**) qRT-PCR analyzed the expression of FL-SMN, Δ7-SMN, and total SMN mRNA in iMNs. *GAPDH* was used as the internal reference. Bars represent the mean ± (SEM); *n* = 3. Ns, not significant. ** *p* < 0.01. *** *p* < 0.001. (**E**) The SMN protein expression levels in hiMNs, SMA-iMNs, and targeted-deletion iMNs (6-iMNs and 18-iMNs). β-actin was used as the internal reference. (**F**) Values for each iMNs were normalized to β-actin protein levels. Data shown indicate mean ± SD. * *p* < 0.05. ** *p* < 0.01. (**G**) Immunostaining of SMN in iPSCs derived iMNs. The red fluorescence showed SMN protein, and the gems were pointed out with white arrows. The green fluorescence indicated ISL1. DAPI was used to visualize the nucleus. ISL1, ISLET1. (**H**) The number of gems detected was higher in the hiMNs, 6-iMNs, and 18-iMNs compared with SMA-iMNs. Data shown indicate mean ± SD. ** *p* < 0.01. *** *p* < 0.001. **** *p* < 0.0001 (**I**) MNs were treated with DMSO or 10 μM camptothecin for 21 h and assayed with TUNEL to mark apoptotic cells. DAPI was used for nuclear staining. ISL1, ISLET1. (**J**) Quantification of TUNEL positive iMNs to total cells after camptothecin treatment. Data shown indicate mean ± Sd. *n* = 3. * *p* < 0.05. ** *p* < 0.01.

Although the exon 7 inclusion rate in targeted-deletion iPSC-derived iMNs was lower than that in hiMNs, it was obviously higher than in SMA-iMNs (Figure 5A). RT-qPCR confirmed that FL-SMN mRNA levels in 6-iMNs and 18-iMNs were significantly higher than in SMA-iMNs (Figure 5B). The total SMN mRNA levels were similar in each group (Figure 5C). For the Δ7-SMN mRNA level, 18-iMNs, the clone with only one edited ISS-N1 copy-derived iMNs, was not different from SMA-iMNs (Figure 5D). Moreover, the cell lysates of ChAT+ iMNs were harvested to assess SMN expression. SMN protein levels in 6-iMNs and 18-iMNs were significantly higher than those in SMA-iMNs and similar to those in hiMNs (Figure 5E,F). Immunostaining of SMN in iPSCs derived iMNs showed that the number of SMN gem structures was significantly increased in 6-iMNs and 18-iMNs and was more than that in SMA-iMNs (Figure 5G,H). Shi-Yan et al. found that the loss of SMN protein leads to the hyperactivity of ER stress and results in MN apoptosis [6]. All iMN cell lines were treated with 10 μM camptothecin, a compound that induces ER stress, for 21 h to investigate whether the increased SMN protein in targeted-deletion clones reduced MNs sensitivity to ER stress. The treated cells were TUNEL stained to evaluate the extent of camptothecin-induced apoptosis. The apoptosis rate of SMA-iMNs was significantly higher than those of hiMNs, 6-iMNs, and 18-iMNs (Figure 5I,J). Therefore, SMN expression was restored in the targeted-deletion iPSC-derived MNs, and SMA MNs degeneration was alleviated.

## 3. Discussion

In this study, precise PE-mediated deletion of the core of the antisense target of ISS-N1 is a promising therapeutic strategy for SMA. In iPSCs and their derived MNs, the inclusion rate of *SMN2* exon 7 and FL-SMN expression was restored once the core of the antisense target of ISS-N1 was targeted for deletion. Notably, MNs derived from targeted-deletion iPSCs could effectively resist ER stress-induced apoptosis.

This new therapeutic strategy has several features and advantages. First, gene manipulation of an intronic element is safer than gene editing of an exon splicing element, which may introduce pathogenic gene mutations in a coding sequence. Second, although artificially engineered nucleases have greatly improved the efficiency of genome editing, homology-directed repair (HDR)-mediated mutation replacement remains difficult in human cells, especially iPSCs without selection markers [22,23,24]. The efficiency of nonhomologous end joining (NHEJ) is much higher than that of HDR [25,26,27]. Therefore, Jin-jing et al. described Cas9-mediated splicing regulatory element disruption with 54 analyzed clones and 13 clones with NHEJ. However, NHEJ, which is highly error-prone, yields a remarkable number of clones (~30.77%) without splicing modulation. That is, FL-SMN expression is improved in only 16.67% of clones, and 7.41% are byproducts [16]. PE is an error-free editing system that can be accurately repaired using RT. Here, in situ targeted-deletion of *SMN2* ISS-N1 in SMA-iPSCs is achieved with a high efficiency of 7/24. Third, no exogenous donor template is needed to achieve accurate disruption via our PE-mediated disruption strategy. Lastly, although the strategy was applied to only one splicing regulatory element, PE is less limited by PAM than by CRISPR/Cas9 [17,28]; theoretically, our strategy applies to other splicing regulatory elements.

The level of SMN transcript, SMN protein expression and location, and the ability to reduce MN sensitivity to ER stress were rescued in targeted-deletion iMNs. Unexpectedly, the level of FL-SMN transcript was inconsistent with the copies of targeted-deletion *SMN2* in 1527N-6 and 1527N-18 (Figure 3C and Figure 5B). More targeted-deletion clones need to be analyzed. The phenotypical of iMNs derived from SMA-iPSCs were different from hiPSCs. Lin et al. reported that the neurite outgrowth was suppressed in the SMA-iPSCs derived MNs, and also found a significant difference in neurite outgrowth between MNs derived from iPSCs of SMA III type and SMA I type in long-term cultures [29]. SMA-iPSCs exhibited a reduced capacity to form MNs [30]. In this study, we found that the SMA-iMNs could hardly survive at low density during MN maturation. Longer-term culture of MNs and a more comprehensive evaluation will be investigated in our further studies.

Therapeutic approaches for SMA have remarkably progressed. For example, small molecules [31,32] or ASOs [15,33,34,35] are used to modulate *SMN2* splicing, increase SMN2-derived FL-SMN, and improve SMN protein levels through the delivery of an exogenous SMN gene by adeno-associated virus (AAV) serotype 9 (AAV9) [36,37]. Risdiplam, Spinraza, and onasemnogene abeparvovec, which are drugs based on these approaches, have been approved by the US Food and Drug Administration (FDA) for SMA treatment [32,38,39]. ASOs can potentially treat many diseases, such as neurodegenerative diseases and diabetes [40,41]. Spinraza (nusinersen), an ASO that blocks ISS-N1, is the first approved SMA therapeutic drug for clinical application. However, it cannot permanently restore the SMN protein because it has a short half-life and requires repeated intrathecal injections. Risdiplam (Evrysdi), a recently FDA-approved oral treatment for SMA, needs to be taken daily. Notably, our strategy avoided the continual administration of ASOs or daily medication by disrupting ISS-N1. Another promising approach is gene therapy, which typically relies on viruses to deliver therapeutic genes into cells. In 2019, the FDA approved AA9 carrying the SMN gene expression cassette. For example, onasemnogene abeparvovec (Zolgensma) is a single-dose gene replacement therapy for SMA. Unfortunately, viral correction has limitations in that it cannot maintain therapeutic gene expression efficiently, and repeated injection of the virus is not feasible [42,43].

In summary, this study was the first to use PE for accurately modifying ISS-N1 of *SMN2*. The targeted-deletion ability of PE in iPSCs was validated, and splicing regulatory elements were efficiently disrupted via PE. SMN expression was significantly restored in targeted-deletion clones. iMNs derived from these clones could resist ER stress-induced apoptosis. Therefore, PE could be used to modify splicing regulatory elements and applied to gene therapy for SMA and other genetic diseases.

## 4. Materials and Methods

### 4.1. PE Design and Construction

pegRNAs were designed according to the target site sequence; two different PBS lengths (13 and 15 bp) with three different RT template lengths were also designed. For pegRNA construction, an expression vector containing nicking sgRNA and pegRNA under the control of the U6 promoter was synthesized by Sangon Biotech. This vector was digested with *Bbs I* (New England Biolabs, Ipswich, MA, USA), gel purified, and ligated with the annealed complementary PBS and RT template oligos using T4 DNA ligase (Thermo Fisher Scientific, Waltham, MA, USA). Six expression plasmids were constructed by combining the different lengths of the PBS and RT templates: 1319N, 1324N, 1327N, 1519N, 1524N, and 1527N. Corresponding pegRNAs without nicking sgRNA were also constructed: 1319, 1324, 1327, 1519, 1524, and 1527. The plasmid constructs were confirmed by Sanger sequencing. The pCMV-PE2 expression vector was provided by David R. Liu (Addgene plasmid #132775; https://www.addgene.org/132775/, accessed on 23 Novenber 2019; Watertown, MA, USA). All the oligo sequences are listed in Table S1.

### 4.2. PE Efficiency Evaluation

HEK-293T cells were transfected using jetPRIME (Polyplus-transfection, Strasbourg, Alsace, France) in accordance with the manufacturer’s instructions. When the confluency of cells seeded on six-well plates reached approximately 70%, the cells were transfected with 1500 ng of pCMV-PE2 plasmid and 500 ng of pegRNA. In this experiment, 12 plasmids of pegRNAs were transfected with different lengths of PBS and RT templates: six plasmids with nicking sgRNA (1319N, 1324N, 1327N, 1519N, 1524N, and 1527N) and six plasmids without nicking sgRNA (1319, 1324, 1327, 1519, 1524, and 1527). Genomic DNA was extracted from the transfected cells 72 h after transfection. The region encompassing the targeted locus was PCR amplified using Phanta^®^ Max SuperFidelity DNA polymerase (Vazyme, Nanjing, China) and detected by Sanger sequencing and targeted deep sequencing. The primers used for PCR and targeted deep sequencing are listed in Supplementary Table S1. PCR amplicons were sequenced using an Illumina Miseq (2 × 300) platform at Sangon Biotec, Shanghai, China. The reads with targeted-deletion (sequence: GTAAGTTTATG) and lengths more than 200 nt were counted. The editing efficiency (Figure 1D) was calculated as the ratio of the summation reads to the total reads.

### 4.3. Gene Targeting and PCR Detection of Targeted-Deletion Clones

The SMA-iPSCs used in this study were described previously [20]. They were dissociated via TrypLE Select (Life Technologies, Grand Island, NY, USA) at 37 °C for 5 min. Single cells were counted and resuspended in 100 µL of the reagent from Human Stem Cell Nucleofector Kit 2 (Lonza, Alpharetta, GA, USA) for nucleofection by using Nucleofector II (Lonza, Alpharetta, GA, USA) set at B016 program. Then, 6 µg of pCMV-PE2 plasmid and 2 µg of pegRNA plasmid (1527N or 1327N) were used to transfect 1 × 10^6^ cells. The transfected cells were cultured on Matrigel (Corning, NY, USA)-coated wells in mTeSR1 (STEMCELL Technologies, Vancouver, Canada). After 2 days, the cells were dissociated into single cells and counted. Subsequently, 500 cells were seeded in a 60 mm culture dish with an mTeSR1 medium supplemented with 10% CloneR (STEMCELL Technologies, Vancouver, Canada). After 14 days, clones were mechanically selected, expanded, and identified through PCR and Sanger sequencing of the modified targeted genome sequences.

### 4.4. Detection of Potential Off-Target Sites

Potential off-target sites were searched using the CCTop-CR ISPR/Cas9 target online predictor (https://cctop.cos.uni-heidelberg.de:8043/, accessed on 20 July 2020) [21]. The top nine sites for pegRNA (Table S2) and the top ten sites of nicking sgRNA (Table S3) were selected for detection. Genomic DNA was extracted from SMA-iPSCs, 1527N-6, and 1527N-18, and potential off-target sites were amplified through PCR and subjected to Sanger sequencing. Off-target effects on gene-edited clones were evaluated by examining their indels. Primer sequences are listed in Table S4.

### 4.5. iPSCs Differentiation into MNs

iPSCs were differentiated into MNs in accordance with previously described methods [20]. Briefly, different media with their corresponding factors were used at various stages, as shown in the differentiation method flow in Figure 4A. An MN-induced medium was composed of DMEM/F12 and a neurobasal medium at 1:1, with 0.5 × N2, 0.5 × B27, 0.1 mM ascorbic acid (Sigma-Aldrich, St. Louis, MO, USA), and 1 × Glutamax. The combination of CHIR99021, DMH1, SB431542, retinoic acid (RA; Sigma-Aldrich, St. Louis, MO, USA), purmorphamine (Pur), valproic acid (Sigma-Aldrich, St. Louis, MO, USA), and DAPT (all others from Selleckchem, Houston, TX, USA) was added to the medium as per the differentiation method. iMNs were characterized via the immunofluorescence staining of surface markers. The primary antibodies used were anti-OLIG2 (Merck Millipore, Watford, UK), anti-SMI32 (BioLegend, San Diego, CA, USA), anti-ISL1 (Merck Millipore, Watford, UK), and anti-ChAT (Merck Millipore, Watford, UK). DAPI was used for nuclear staining. Stained cells were examined, and images were photographed using a fluorescence microscope. To analyze the number of gems, a previously described protocol was used [20]. At least 50 nuclei per slice were surveyed via a randomly selected field from each slice by independent researchers who were blind to the status of the cell genotype. The details of the primary antibodies are presented in Table S5.

### 4.6. Reverse Transcription PCR (RT-PCR) and Quantitative RT-PCR (qRT-PCR) Analysis

Total RNA was isolated using a TRIzol reagent (Sigma-Aldrich, St. Louis, MO, USA #T9424), and potential residual DNA was eliminated using a gDNA wiper mix (Vazyme, Nanjing, China). RNA samples were then reverse transcribed into cDNA using a HiScript II first-strand cDNA synthesis kit (Vazyme, Nanjing, China). RT-PCR was performed with the primers F (exon 6) and R (exon 8). The obtained products were separated using 12% polyacrylamide gel. The gray values of the DNA bands were analyzed using ImageJ, and the inclusion rates were calculated as the ratio of the FL-SMN band gray value to the grayscale value of FL-SMN plus Δ7-SMN.

qRT-PCR was performed using a HiScript II one-step qRT-PCR SYBR Green kit (Vazyme, Nanjing, China) in accordance with the manufacturer’s instructions on a Bio-Rad CFX96 Touch q-PCR system. Two pairs of previously reported primers were used to detect FL-SMN and Δ7-SMN transcripts. GAPDH was used as an endogenous control.

### 4.7. Western Blot

The cells were lysed with RIPA lysis buffer containing 1× phenylmethanesulfonyl fluoride (PMSF, 1 mM) and 1% protease-inhibitor cocktail on ice for 5 min. The lysates were collected, sonicated, heated at 95 °C for 10 min, and measured with a BCA protein assay (Thermo Fisher Scientific, Waltham, MA, USA). Protein samples (15 μg) from each group were subjected to 10% polyacrylamide gel electrophoresis and transferred onto polyvinylidene fluoride membranes. After being blocked with 5% nonfat milk in PBST (0.1% Tris-buffered saline with Tween-20), the membranes were incubated with mouse anti-SMN (BD Transduction Laboratories, Franklin Lakes, NJ, USA) and mouse anti-β-actin (Sigma-Aldrich, St. Louis, MO, USA) at 4 °C overnight. Then, they were washed with PBST, incubated with anti-mouse horseradish peroxidase-conjugated secondary antibodies (Abcam, Cambridge, UK) at 20–25 °C for 1 h, and visualized via enhanced chemiluminescence by using an ECL detection kit (Thermo Fisher Scientific, Waltham, MA, USA). The details of the antibodies are presented in Table S5.

### 4.8. Statistical Analysis

Data were analyzed using GraphPad Prism (v8.3.0; GraphPad Software, La Jolla, CA, USA). All values were presented as mean ± standard error of the mean. Data among three or more groups were compared via one-way ANOVA.

## Data Availability

All data supporting the reported result in this study can be found in the Supplementary Materials.

## Supplementary Materials

The following supporting information can be downloaded at: https://www.mdpi.com/article/10.3390/ijms23147941/s1.
