# Supplementary material for: Targeted-Deletion of a Tiny Sequence via Prime Editing to Restore SMN Expression
Source: Int J Mol Sci. 2022 Jul 19;23(14):7941. doi: 10.3390/ijms23147941 (PMC9317564; doi:10.3390/ijms23147941)
Supplement: Supplementary file 1 [file ijms-23-07941-s001.zip › ijms-1799316-supplementary.pdf]

Supplementary Material

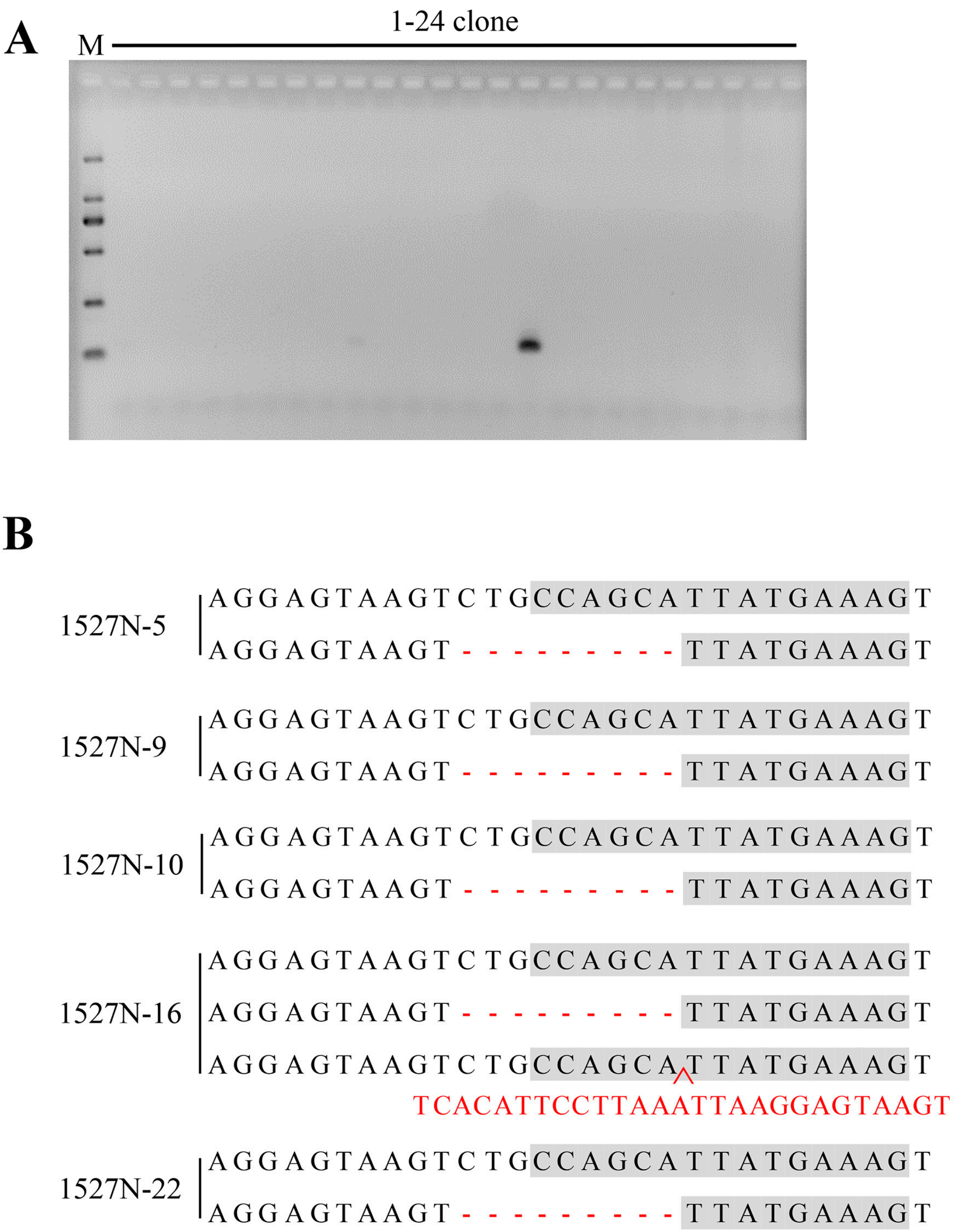

Figure S1. Targeted-deletion and screening. (A) Twenty-four candidate mono-clones of 1327N were picked, followed by genomic DNA extraction and PCR analysis using specific primer. The targeted-deletion obtained a 121 bp band. M, DL2000 DNA ladder. (B) T-A cloning and sequencing of the positive clones of 1527N. The deletions are indicated by a red dashed line. The insertions are indicated by red characters. The column on the right indicates the percent of the relevant genotype in total sequencing reads. WT, wild-type. Δ, deletion. "+", insertion.

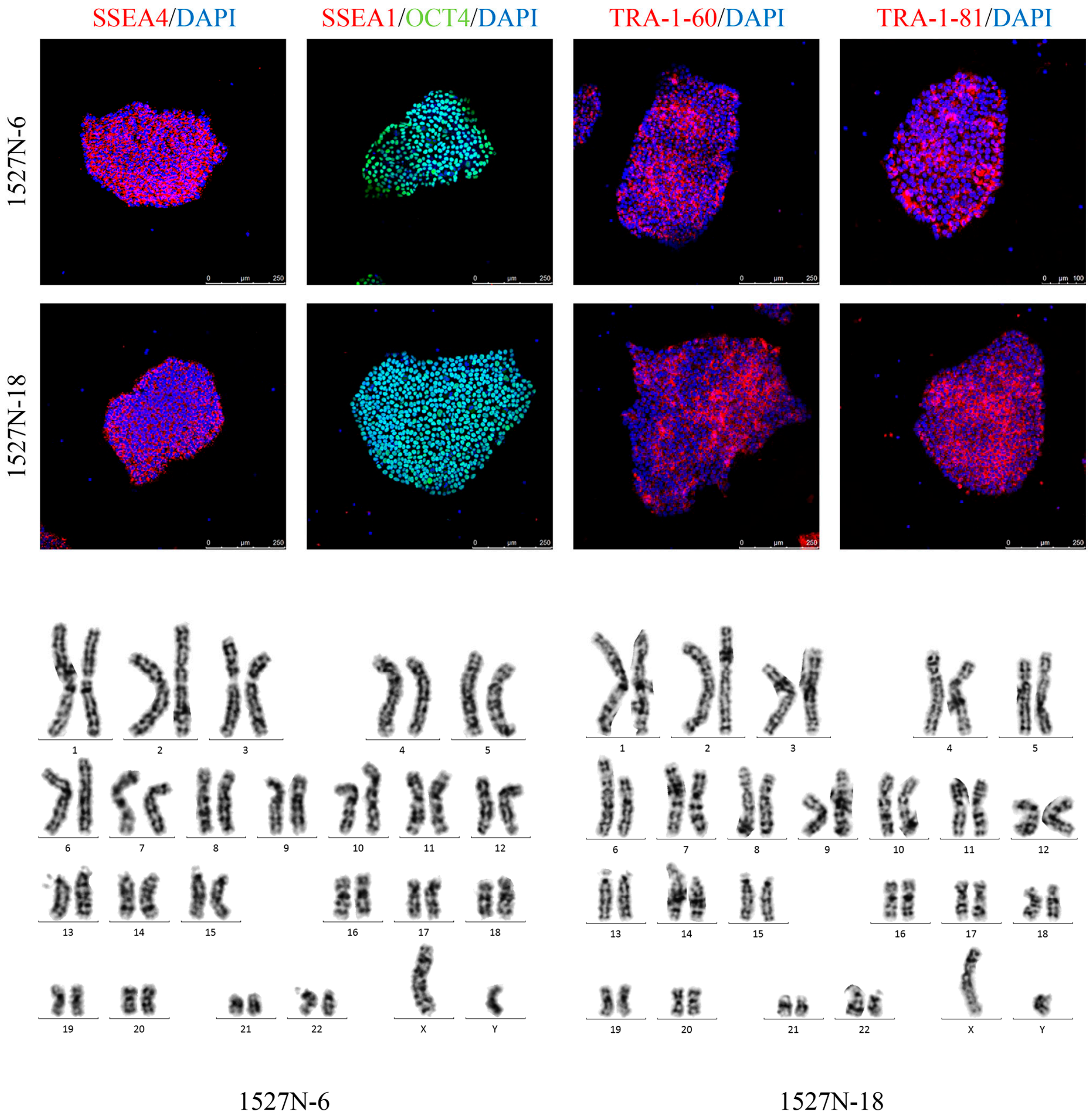

Figure S2. Characterization of the targeted-deletion iPSCs clones. Expression of pluripotency markers in targeted-deletion 1527N-6 and 1527N-18 cell line were detected by immunocytochemistry and karyotype. DAPI indicate the total cell present in the image. The targeted-deletion iPS cell lines are maintain the pluripotency of stem cell and the normal karyotype.

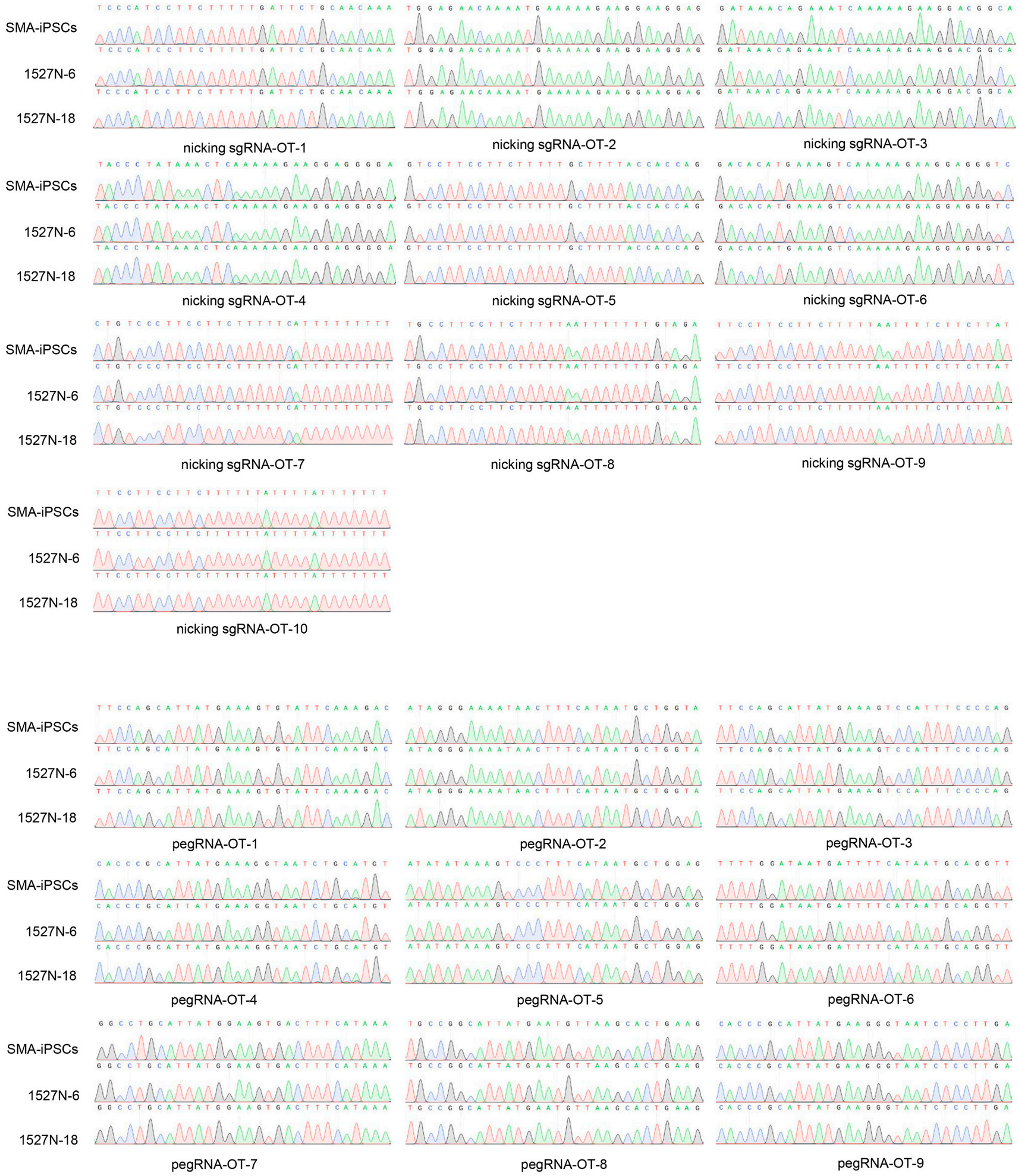

Figure S3. Sanger sequencing of the top ten potential off-target sites of nicking sgRNA and top nine sites for pegRNA after targeted-deletion. We analyzed the initial patient cells and the clone 1527N-6 and 1527N-8. No indels at the sites were found.

Table S1. The oligos and primers used in this paper.

nicking sgRNA target sequence	Forward	5'-CACCgacaaaatcaaaaagaagga-3′
	Reverse	5′-AAACtccttctttttgattttgtc-3′
Oligos used for pegRNAs construction	1319F	5'-5GTGCcttaaattaaggagtaagtttatgaaagtgaa-3'
	1319R	5'-AAAAttcactttcataaacttactccttaatttaag-3'
	1324F	5'-GTGCcattccttaaattaaggagtaagtttatgaaagtgaa-3'
	1324R	5'-AAAAttcactttcataaacttactccttaatttaaggaatg-3'
	1327F	5′-GTGCtcacattccttaaattaaggagtaagtttatgaaagtgaa-3′
	1327R	5′-aaaattcactttcataaacttactccttaatttaaggaatgtga-3′
	1519F	5'-GTGCcttaaattaaggagtaagtttatgaaagtgaatc-3'
	1519R	5'-AAAAgattcactttcataaacttactccttaatttaag-3'
	1524F	5'-GTGCcattccttaaattaaggagtaagtttatgaaagtgaatc-3'
	1524R	5'-AAAAgattcactttcataaacttactccttaatttaaggaatg-3'
	1527F	5′-GTGCtcacattccttaaattaaggagtaagtttatgaaagtgaatc-3′
	1527R	5′-aaaagattcactttcataaacttactccttaatttaaggaatgtga-3′
PCR for identify targeted deletion clone	specific primer F1	5′-cattccttaaattaaggagtaagtttatg-3′
	specific primer R1	5′-cctttcaactttctaacatctgaact-3′
PCR for T-A cloned of mono-clones	Forward	5′-tctcaagtgatccccctacct-3′
	Reverse	5′-gtttcttccacacaaccaacc-3′
PCR for targeted deep sequencing	Forward	5′- aaaatgtcttgtgaaacaaaatgc -3′
	Reverse	5′-cctttcaactttctaacatctgaact-3′

RT-PCR for exon 7 inclusion rates	Forward	5′-GCTGATGCTTTGGGAAGTATGTTA-3′
	Reverse	5′-GTCTGATCGTTTCTTTAGTGGTGTC-3′
qRT-PCR for total SMN mRNA	Forward	5′-GCTGATGCTTTGGGAAGTATGTTA-3′
	Reverse	5′-GTCTGATCGTTTCTTTAGTGGTGTC-3′
qRT-PCR for Full-length SMN	Forward based on exon 6	5′-GCTGATGCTTTGGGAAGTATGTTA-3′
	Reverse based on exon 7	5′-CACCTTCCTTCTTTTTGATTTTGTC-3′
qRT-PCR for Δ7 SMN	Forward based on exon 5/6	5′-TGGACCACCAATAATTCCCC-3′
	Reverse based on exon 6/8	5′-ATGCCAGCATTTCCATATAATAGCC-3′
qRT-PCR for GAPDH	Forward	5′-GACTTTGCTTTCCTTGGTCAGG-3′
	Reverse	5′-AGTCTGGCTTATATCCAACACTTCG-3′

Table S2. Potential off-target sites of pegRNA.

sequence	mismatches	locus
TGAATACACTTTCATAATGC	4MMs[1:2:3:6] with pegRNA	chr12:129455147-129455169
GAAAATAACTTTCATAATGC	3MMs[3:5:7] with pegRNA	chr7:142217490-142217512
GAAATGGACTTTCATAATGC	3MMs[3:6:7] with pegRNA	chr7:126088251-126088273
CAGATTACCTTTCATAATGC	3MMs[1:7:8] with pegRNA	chrX:35976377-35976399
TAAAGTCCCTTTCATAATGC	4MMs[1:3:5:8] with pegRNA	chr2:222190141-222190163
GATAATGATTTTCATAATGC	4MMs[3:5:7:9]with pegRNA	chr5:17214384-17214406
GAAAGTCACTTCCATAATGC	3MMs[3:5:12] with pegRNA	chr15:78855498-78855520
GTGCTTAACATTCATAATGC	4MMs[2:4:7:10] with pegRNA	chrX:107726513-107726535
GAGATTACCCTTCATAATGC	3MMs[7:8:10] with pegRNA	chr5:10647841-10647863

The off-target sites were predicted by the CCTop-CRISPR/Cas9 target online predictor. The mismatch is indicated by colored letters.

Table S3. Potential off-target sites of nicking sgRNA.

sequence	mismatches	locus
TGCAGAATCAAAAAGAAGGA	3MMs[1:2:5] with nicking sgRNA	chr4:173210765-173210787
AACAAAATGAAAAAGAAGGA	2MMs[1:9] with nicking sgRNA	chr1:207699639-207699661
ACAGAAATCAAAAAGAAGGA	4MMs[1:2:3:4] with nicking sgRNA	chr20:48718502-48718524
TATAAACTCAAAAAGAAGGA	3MMs[1:3:7] with nicking sgRNA	chr4:15535358-15535380
GGTAAAAGCAAAAAGAAGGA	3MMs[2:3:8] with nicking sgRNA	chr2:170521750-170521772
ATGAAAGTCAAAAAGAAGGA	4MMs[1:2:3:7]with nicking sgRNA	chr2:18765858-18765880
AAAAAAATGAAAAAGAAGGA	3MMs[1:3:9] with nicking sgRNA	chr4:104654918-104654940
AAAAAAATTAAAAAGAAGGA	3MMs[1:3:9] with nicking sgRNA	chr7:129631378-129631400
AAGAAAATTAAAAAGAAGGA	3MMs[1:3:9] with nicking sgRNA	chr3:107870720-107870742
AATAAAATAAAAAAGAAGGA	3MMs[1:3:9] with nicking sgRNA	chr2:54380149-54380171

The off-target sites were predicted by the CCTop-CRISPR/Cas9 target online predictor. The mismatch is indicated by colored letters.

Table S4. Primers used in off-target analysis.

Site	Forward primer	Reverse primer	Expected product size, bp
nicking sgRNA-OT-1	ATCTAGTGAGGGTACAGGCAGG	GTCACCTTTGCACTTTTGCAC	899
nicking sgRNA-OT-2	AACATGGGGACAGGGACTTC	TCTTGGCTGAAACAGCTCGC	692
nicking sgRNA-OT-3	CGGCTCACCGTGAAGTTCTA	ATGGTCATGCTTGTTAAATGTTGGT	767
nicking sgRNA-OT-4	GATGCACAGTGGTACCCGAA	GCAGGGACCCTATTGCCATT	612
nicking sgRNA-OT-5	CCGCCCTTCTCTTCACCTTA	GAGACTTGCGACTCGTGCTC	696
nicking sgRNA-OT-6	CATCCGAGCCCTGATGAGAC	ATCTGGCTCTACGTGTCCCT	897
nicking sgRNA-OT-7	TCTATGAAGACCTCAAAAGATGCA	TCCTTACCTCCTTCTCCCCAC	403
nicking sgRNA-OT-8	GCATTTCGAAGTTTGACCTCCTT	AACACTGAATAGGCTTCTCCCTG	682
nicking sgRNA-OT-9	GTAGGTTGCACCAATTTTTATCCCT	ACCCACTGAGATACGATGATGC	604
nicking sgRNA-OT-10	TGAGCCGAGATAGCACCATTG	GGGGAGAGCGTATCAGCACA	733
pegRNA-OT-1	AGCCATCCACAAGTGCCATTA	CCCAGTGAAATGGTGCCCTC	800
pegRNA-OT-2	AGTTCTCTCTTGACCCTCTTCCTC	TTGTTCCCACTAACCAACCTCTC	588
pegRNA-OT-3	TTATCTCTACCTGGTTCCCCCT	TTCACAAAGTTCAAAGCCTGG	587
pegRNA-OT-4	GTACTGTGTACTGGTGCCAAGA	CACAAATACAGGGCACGTCAA	879
pegRNA-OT-5	ATGAAGGTCTGCCTTTGGGG	ATGGGCTTGGAAAGGTAGCC	390
pegRNA-OT-6	TCACACTCGGGTGACACTAC	GGTTGCCAAAATGTTACCTCTCAA	841
pegRNA-OT-7	GGGGGTGGGGAAAGAAATAC	GCTTCCACCTTTTGCCTATTG	869
pegRNA-OT-8	CCTCACCCCATTCCATTGCT	CAGAGACCTGGGCACTTCTG	600
pegRNA-OT-9	ATATTTGGGGGCAGTGGGTG	CCTTTAACCGATGGTGGGCT	676

Table S5. Sources and applications of antibodies.

Antibody	Source	Catalogue	Application
OCT4	Abcam	ab181557	1:100 (IF)
NANOG	Abcam	ab109250	1:100 (IF)
SSEA-1	Merck Millipore	SCR001	1:100 (IF)
SSEA-4	Merck Millipore	SCR001	1:100 (IF)
TRA-1-60	Merck Millipore	SCR001	1:100 (IF)
TRA-1-81	Merck Millipore	SCR001	1:100 (IF)
OLIG2	Merck Millipore	#AB9610	1:500 (IF)
Islet-1	Merck Millipore	#AB4326	1:100 (IF)
SMI32	BioLegend	# 801701	1:100 (IF)
HB9	Sigma-Aldrich	HPA071717	1:100 (IF)
ChAT	Merck Millipore	#AB144P	1:100 (IF)
SMN	BD Transduction Laboratories	610646	1:100 (IF), 1:10,000 (WB)
β-actin	Sigma-Aldrich	A5441	1:10,000 (WB)
anti-mouse horseradish peroxidase-conjugated secondary antibodies	Abcam	ab6789	1:10,000 (WB)

IF, immunofluorescence; WB, western blot.
